# Discovering non-random segregation of sister chromatids: the naïve treatment of a premature discovery

**DOI:** 10.3389/fonc.2012.00211

**Published:** 2013-02-01

**Authors:** Karl G. Lark

**Affiliations:** Department of Biology, University of UtahSalt Lake City, UT, USA

**Keywords:** non-random segregation, sister chromatid, stem cell, mouse, radioautography

## Abstract

The discovery of non-random chromosome segregation (**Figure 1**) is discussed from the perspective of what was known in 1965 and 1966. The distinction between daughter, parent, or grandparent strands of DNA was developed in a bacterial system and led to the discovery that multiple copies of DNA elements of bacteria are not distributed randomly with respect to the age of the template strand. Experiments with higher eukaryotic cells demonstrated that during mitosis Mendel’s laws were violated; and the initial serendipitous choice of eukaryotic cell system led to the striking example of non-random segregation of parent and grandparent DNA template strands in primary cultures of cells derived from mouse embryos. Attempts to extrapolate these findings to established tissue culture lines demonstrated that the property could be lost. Experiments using plant root tips demonstrated that the phenomenon exists in plants and that it was, at some level, under genetic control. Despite publication in major journals and symposia (Lark et al., 1966, 1967; Lark, 1967, 1969a,b,c) the potential implications of these findings were ignored for several decades. Here we explore possible reasons for the pre-maturity (Stent, 1972) of this discovery.

## INTRODUCTION

**FIGURE 1:**
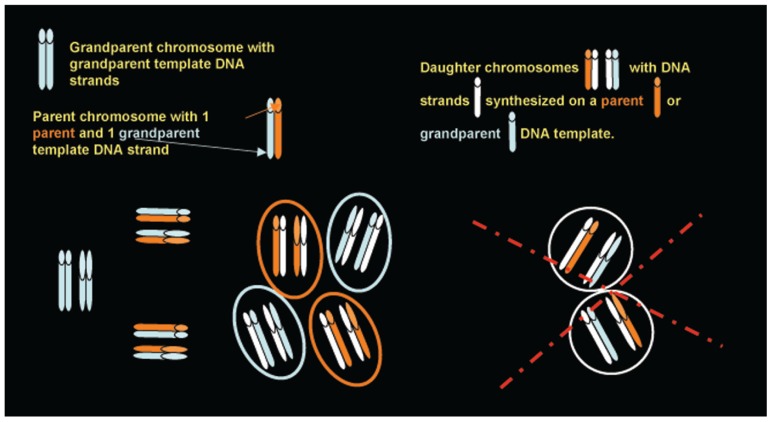
**Non-random segregation of chromosomes synthesized on DNA templates of different ages**. Granddaughter cells (circled) contain chromosome sets synthesized either on grandparent DNA templates or on parent DNA template, but do not (**X**) receive sets that are mixtures of chromosomes synthesized some on grandparent- others on parent-templates.

In 1966, Richard Consigli, Harish Minocha and I published a paper in Science entitled “Segregation of sister chromatids in mammalian cells” (Lark et al., 1966). The first sentence of the abstract read as follows: “Segregation of sister chromatids in embryonic mouse cells in primary tissue culture is not random.” In so doing, we reported the unexpected existence of non-random mitotic segregation of eukaryotic chromosomes in stem cells, the focus of this book. Non-random segregation in mouse cells was discovered as a consequence of analyzing bacterial DNA replication and segregation. During the next few years (1967–1969) we demonstrated the phenomenon in plant cells as well.

Our results are an example of the discovery of a phenomenon for which an appropriate hypothesis was lacking at the time; possibly an anomaly because it did not lend itself to any existing body of information in either a supportive or contradictory role. It was *data driven science* – and in some sense, premature (see Stent, 1972). This brief memoir traces the sequence of events leading to the discovery of non-random replication and describes the scientific context at that time: what we knew and what we did not know (or even suspect) may explain the pre-maturity that often becomes associated with data driven science.

## THE DISCOVERY OF NON-RANDOM SEGREGATION

In 1963, we began a series of experiments on DNA replication (and eventually segregation) in bacteria (Lark et al., 1963; Lark and Bird, 1965; Lark, 1966b,c). A decade had passed since the annunciation of the structure of DNA (Watson and Crick, 1953) during which ingenious experiments had: (i) verified that structure (Josse et al., 1961); (ii) demonstrated the existence of semi-conservative replication in eu- and pro-karyotes (Taylor et al., 1957; Meselson and Stahl, 1958); and (iii) suggested a mechanism for regulating the initiation of DNA synthesis in bacteria (Jacob et al., 1963).

Four experimental tools were essential to these results: pulse chase as a technique for *in vivo* analysis of sequential intra cellular events (Roberts, 1964); autoradiography of tritiated thymidine labeled DNA (Taylor et al., 1957; Painter, 1958); density labeling of DNA (^15^N: Meselson and Stahl, 1958; or 5-Bromo-uridine: Lark et al., 1963); and the use of conditional lethal mutations to dissect intracellular bacterial processes (Epstein et al., 2012).

The cell biology of bacterial growth also had been analyzed during that decade (Schaechter et al., 1958), demonstrating, among other things, that the cellular content of RNA and DNA changed when bacteria grew at different growth rates in different media. In poor media (slower growth rates), the content of RNA and DNA were lower than during more rapid growth in richer media. Our experiments utilized a strain of *Escherichia coli*, 15T^-^, which could contain either two separate replicating chromosomes per cell, or only one depending on the growth rate determined by different nutrients. When grown rapidly (in glucose), the two chromosomes would replicate at the same time, whereas at somewhat less rapid rates of growth (succinate) first one would be replicated and then the other (Lark and Lark, 1965; Lark, 1966a). As far as could be determined the two chromosomes were biologically identical, since when grown very slowly (acetate) cells contained only one chromosome, but could regenerate the two chromosome content if transferred to a medium promoting faster growth.

In order to establish the chromosome content of 15T^-^ cells grown at these different rates, we labeled cells with a pulse of tritiated thymine and then grew them in non-radioactive medium for different periods (chase) and plated them at different times onto non-radioactive nutrient agar to allow the development of microcolonies. Autoradiography of either cells or the microcolonies derived from these cells established the number of radioactive DNA units labeled by the pulse (Lark and Bird, 1965). We expected that after a chase period, chromosomes would be distributed randomly – i.e., these cells each containing two chromosomes would contain two radioactive chromosomes, one radioactive and one non-radioactive chromosome, or two non-radioactive chromosomes in frequencies predicted by a binomial distribution. A surprising result was that labeled and unlabeled daughter chromosomes were not distributed randomly into daughter cells. Instead, each daughter received one of each!! This suggested to us that *during DNA segregation, the cells somehow distinguished between apparently identical chromosomes on the basis of the age of their template strands *(Lark, 1966c)*.*

We were curious if such discrimination might occur during eukaryotic somatic growth, but we had not worked with any eukaryotic system. In order to test this we decided to use a cell culture system and approached a colleague, “Dick” Consigli, who was studying polyoma virus grown on tissue cultures of mouse embryo cells (Consigli et al., 1966). In collaboration with Consigli and Minocha, we labeled *primary* cell cultures derived from embryonic tissue with tritiated thymidine and subsequently grew them in non-radioactive medium (“chase”). This serendipitous selection of a primary cell culture yielded dramatic results described in the 1966 Science paper.

**FIGURE 2:**
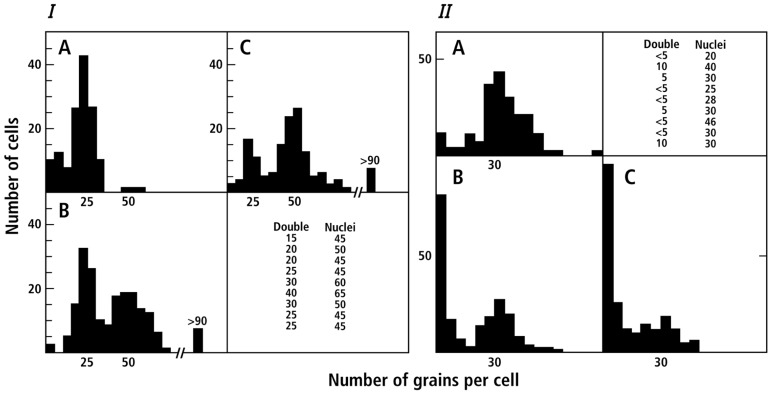
**Non-random segregation of radioactive chromosomes in primary cultures of mouse embryo cells (data taken from Figures 1 and 2 of Lark et al., 1966)**. Distribution of silver grains in autoradiographs of mouse embryo cells: **(I)** Grown as a primary tissue culture for 1, 2, or 3 generations in H3-thymidine (0.025 mc/ml). **(A)** An inoculum of 2.5 × 10^6^ cells per Petri dish (100 mm) was grown for 24 h. **(B)** 1.25 × 10^6^ cells were grown for 48 h. **(C)** 0.63 × 10^6^ cells were grown for 72 h. **(II)** Grown as a primary tissue culture for one generation in H3-thymidine (0.025 mc/ml) and for two subsequent generations in non-radioactive medium (chase). **(A)** 0.3 × 10^6^ cells per Petri dish (35 mm) were grown for 24 h, in medium containing H3-thymidine; **(B)** 0.15 × 10^6^ cells were grown for 24 h as in **(A)**, and then the medium was replaced with non-radioactive medium and cells grown for an additional 24 h; **(C)** 0.08 × 10^6^ cells were grown for 24 h as in **(A)** and then for 48 h in non-radioactive medium. For details see Lark et al. (1966). The solid curves represent the result expected under a null hypothesis of random segregation of equally labeled chromosomes.

**Figure 2** presents the results of labeling the cells continuously for several generations (**Figure 2(I)**) or of a period of radioactive labeling followed by growth in non-radioactive medium (chase **Figure 2(II)**). It was immediately evident that the amount of radioactivity in cells increases or decreases discontinuously in a manner to be expected if the 40 chromosome templates that had incorporated radioactivity remained together.

An unexpected bonus of this cell system was the frequent occurrence of cells with two nuclei that had yet to divide. Radioactivity of such nuclei (grains per nucleus) are also presented in the experiments in **Figures 2(I) and (II)**. After a two to three generation chase many of the two nucleate cells were still radioactive, but only one nucleus was heavily labeled. Segregation was not random in these mouse cells.

We soon discovered that established tissue culture lines (HeLa or CHO) had lost this property (Lark et al., 1966). Had we begun with established cell lines, we would probably have concluded that the non-random segregation we had documented in bacteria was not a property of somatic mammalian cell division, and we would have abandoned the investigation. Instead, we speculated that the polyploid nature of these established cell lines had obscured the non-random segregation of diploid chromosome sets. Although the distinction between cell lines that had acquired immortality and primary cell lines with programmed longevity was known (Hayflick and Moorhead, 1961; Hayflick, 1965), we had not considered this distinction as a possible explanation for the difference between a primary mouse line and the HeLa or CHO lines.

**FIGURE 3:**
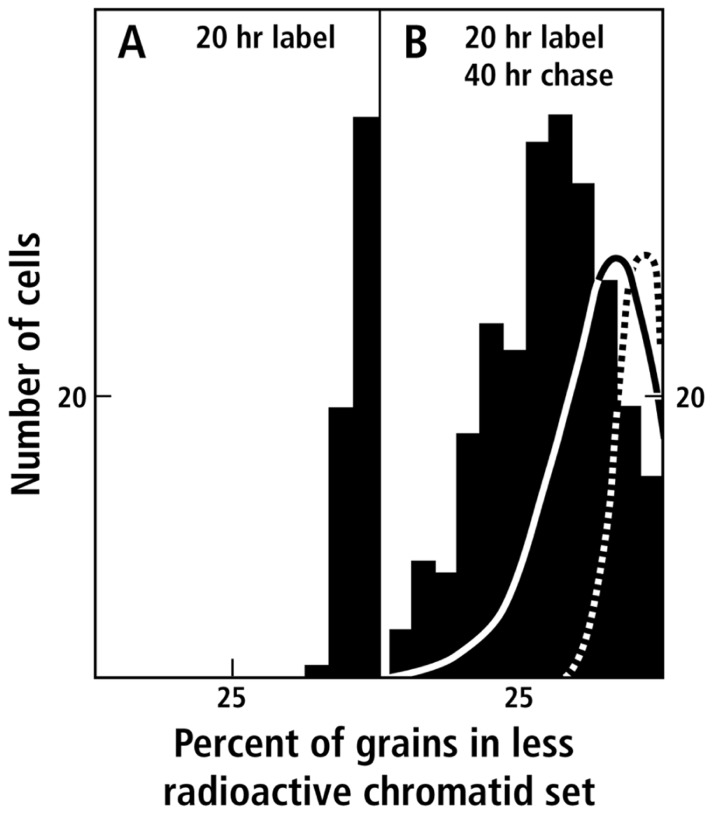
**Segregation of radioactively labeled sister chromatids in *V. faba* root tips (data taken from Figure 3 of Lark, 1967)**. Radioautographs of anaphase or telophase figures in dividing cells were scored for the number of grains over each sister chromatid set. The distribution of cells is presented as a histogram of the number of cells (ordinate) categorized according to the percent radioactivity in the less radioactive member of the cell’s chromatid pair (abscissa): **(A)** After 20 h of labeling with radioactive thymidine; **(B)** after 20 h of labeling with radioactive thymidine followed by 40 h growth in non-radioactive medium. Deviation to the left of 50% indicates the degree of asymmetry. The dashed line indicates the random distribution of 12 radioactive chromatids calculated from the terms of a binomial expansion, assuming no sister chromatid exchange. The solid line is the distribution expected for the random distribution of 12 radioactive chromatids in which sister chromatid exchange results in the redistribution of radioactive material such that on the average 70% of the original chromatid material is not exchanged (for details, see Lark, 1967).

Our desire to avoid possible changes in ploidy during prolonged tissue culture led us to search for alternative preparations in which the non-random segregation could be studied *in vivo.* Autoradiographic analysis of mitosis in plant root tips had been used with great success by Taylor et al. (1957), who elegantly used autoradiography to establish that a chromosome was one DNA molecule that replicated semi-conservatively. We decided to use this system and settled on a pulse-chase protocol in which root tips were first grown for a period in radioactive thymidine (pulse) and then grown in non-radioactive medium for a much longer time (chase). We analyzed root tips of the diploid bean, *Vicia faba* that has 12 easily visualized chromosomes, and in which sister chromatid exchange had been studied in detail by Peacock (1963). By examining anaphase preparations we could compare the amount of radioactivity in the two sets of chromosomes separating after the pulse-chase had been completed. The data (Lark, 1967), corrected for sister chromatid exchange, clearly supported non-random segregation of chromosomes in which “parent template” radioactive DNA was separated from non-radioactive “grandparent template” DNA (**Figure 3**).

To test the idea that polyploidy might obscure non-random segregation we also examined root tips of wheat, *Triticum aestivum* (2*n* = 42) a hexaploid composed of three sets of similar but not identical *homeologous* chromosomes. The results of our pulse-chase experiments were not clear-cut. The difference between chromosome sets in anaphase preparations was less apparent than in *V. faba*, (Lark, 1967), but left open the possibility that there might be a sub population of cells in which segregation was non-random. In contrast, chromosome segregation was clearly non-random in *Triticum boeticum *(4*n* = 28; AAAA) a tetraploid relative of modern wheat. Cytological studies of mitosis in wheat (Feldman et al., 1966) had just been published suggesting that during inter-phase, chromosomes did not completely de-condense and that homeologous sets of chromosomes were not randomly distributed throughout the nucleus thus opening the possibility that segregation of each of the three diploid sets that composed the hexaploid might be regulated autonomously. We therefore decided to further analyze segregation in *Triticum *using genetically different, but related, plants.

**FIGURE 4:**
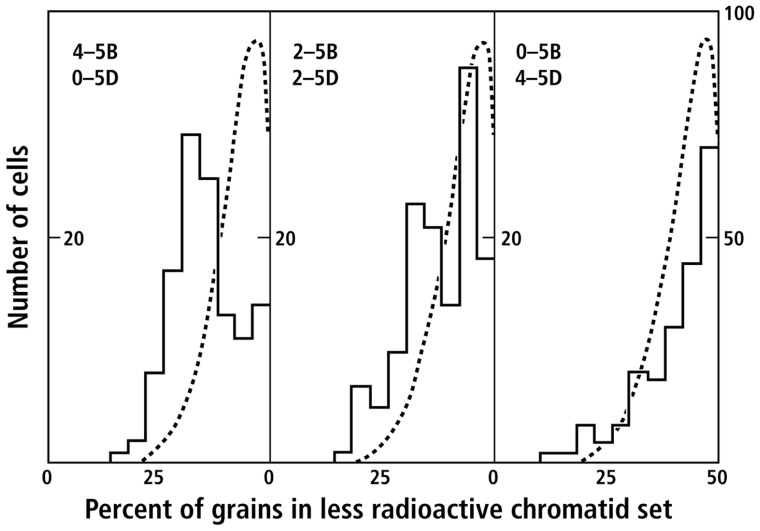
**Sister chromatid segregation in mitotic figures of *T. aestivum*: effects of varying the dosage of chromosomes 5B and 5D (data from Lark, 1969a, Figure 7)**. Histograms of the frequency of cells in which the indicated percentages of grains were observed in the less radioactive chromatid set during anaphase or telophase. Deviation to the left of 50% indicates the degree of asymmetry. The dashed curve represents the expected binomial distribution for random segregation (for details, see Lark, 1969a).

An outstanding achievement of 20th century evolutionary cytogenetics was the research by Hitoshi Kihara (Japan), Earnest Sears (USA), and Nikolai Vavilov (Russia; reviewed by Crow, 1994) that led to an understanding of the origins of hexaploid wheat. Their analysis established that wheat was a hexaploid composed of three diploid sets of similar, but not identical*, homeologous *chromosomes (A, B, and D; 2*n* = 14/set). Their research had produced a number of genetically different *Triticum* lines. We obtained seeds from Sears and in the process learned about the history of wheat and the use of polysomic/nullisomic lines to associate phenotypes with specific chromosomes or portions of chromosomes. In polysomic/nullisomic lines of wheat, particular chromosomes, or arms of chromosomes, of one homeologous set are replaced by extra copies of the same chromosome from another, different, homeologue. For example nulli5B-tetra5D (2-5A:0-5B:4-5D) or nulli5D-tetra5B (2-5A:4-5B:0-5D) lines lack *homeologous* chromosome 5B or 5D, respectively. The results (**Figure 4**) of comparing radioactive segregation in anaphases of cells from pulse-chased root tips from these different lines led to the conclusion that a locus, or loci, on chromosome 5 regulated non-random segregation (Lark, 1969a): 5B promoted non-random segregation; 5D promoted random segregation.

Additional experiments varying the chromosome 5 dosage of different homeologs (*A*, *B*, *or D*) in tetraploid lines of *Triticum* (Lark, 1969a) led to the conclusions that: (i) non-random segregation was a normal process in wheat, (ii) a locus on chromosome 5*D* suppressed non-random segregation, (iii) the magnitude of this effect in the presence of 5B was dose dependent, and (iv) that although 5A alone (e.g., tetra 5A in *T. boeticum*) resulted in non-random segregation, 5A was recessive to 5D in an amphidiploid (AADD = random segregation).

In summary, at the end of 1969 we knew the existence of non-random segregation in three different kingdoms: Eubacteria, Plantae, and Animalia. It appeared that in these systems, Mendel’s second law was abrogated during mitosis of cells *in vivo* or in primary cell culture.

As with most data driven science, our discovery was premature in that we were unprepared for this result. In a lecture at the University of Lille in December 1854, Louis Pasteur noted that “*when collecting data (les champs de l′ observation)* chance favors none but the prepared mind.” Non-random segregation was a most unexpected finding falling on minds conditioned to the random segregation of chromosomes during meiosis. We were unprepared to consider the consequences of separating chromosome sets according to the generation of the template on which new DNA was replicated.

We did not appreciate the full implications of observing the phenomenon in bacteria and plant root tips as well as in cultured cells taken from mouse embryos and failed to explore the evolutionary implication that selection favored such a process. Had we done so, we would have concluded that: (1) maintaining an informational interactive network distributed between multiple chromosomes might be used to discriminate between cells of different generations; (2) in order for that to occur, there should be frequent changes in the chromosomes or their attached proteins, between one generation and the next (imprinting); (3) such changes could provide adaptation (for plants and bacteria) to unforeseen changes in their environment, or using programmed changes in the cell’s environment (e.g., in mammals), it could facilitate morphogenesis or embryogenesis. With these last considerations in mind we might have asked, “What is special about primary mouse embryo cells?,” rather than focus our attention on defects in HeLa and CHO cells.

Significantly two events had occurred in the late 1960s that should have directed my attention to the important role that non-random segregation might play in biological systems: One was the demonstration by my wife Cynthia Lark (Lark, 1968a,b,c) of the methylation of newly synthesized strands of bacterial DNA and the discovery that DNA replication would not proceed *in vivo *if a template strand was not methylated. Most importantly, she collaborated with Werner Arber to demonstrate that host specificity phenotypes were regulated by in vivo methylation of DNA (Lark and Arber, 1970) – i.e., *modification of DNA without changing nucleotide sequence could result in changes of phenotype, an early manifestation of imprinting.*

The other was a visit with Don Brown at the Carnegie Institute of Embryology in Baltimore. When I told Brown about non-random segregation in mouse cells he responded that if this were true it would have extremely important ramifications for developmental biology.

As a cell biologist I had naively concentrated on a cellular and genetic description of the phenomenon, but failed to even speculate on the possible advantages conferred on an organism by this aspect of mitosis. Had I done so, I would have realized that this discovery signaled changes involving chromosomes or closely associated (tightly bound) proteins that differentiated one generation of sister chromatids from another, a realization that should have triggered an attempt to identify such *epigenetic *changes.

## RETROSPECTIVE

This research was interrupted in 1969 by illness and a change in my career to become chair of the Department of Biology at the University of Utah. Research on bacteria continued in my laboratory carried out by post-docs and students, but personal, hands on, experimentation ceased for a period of several years.

The department in Utah was, and still is, united by a common deep interest in evolutionary phenomena and my exposure to this from different faculty, ranging from population ecologists to molecular biologists, returned my thoughts if not my hands to non-random segregation. John Cairns, a friend and colleague, who shared a deep interest in DNA replication, visited for a few weeks in 1972 and 1973, during which we discussed the non-random segregation experiments. Our results interested him and eventually led to his hypothesis on the role of non-random segregation in maintaining template fidelity, consequently reducing the frequency of cancer in populations of rapidly reproducing intestinal epithelial cells (Cairns, 1975). Cairns’ ideas thus were the first formal exposition of the concept that in higher eukaryotes non-random segregation would serve to differentiate between cells of different generations whose function and fate would thenceforth be different.

At that time very little genetic analysis of interactive networks had been carried out. Genetics was still focused on single gene effects. Phenotypes were mostly qualitative and the techniques for analyzing quantitative genetic systems were just beginning to be developed (e.g., Falconer and Mackay, 1996). Today, we are very aware of genome wide interactions and their importance in regulating a multitude of quantitative phenotypes. Thus, the selective advantage of non-random mitotic chromosome segregation in preserving networks of inter-chromosome information now seems evident. Cairns’ ideas focused on the exclusion of deleterious mutations thus maintaining the integrity of stem cells with “healthy” genomes. However, if we invoke the idea of directed epigenetic variation involving multiple chromosomes, non-random segregation can be viewed as a mechanism for preserving cell lineages with useful adaptations. Such lineages could then be maintained throughout development and morphogenesis (Reik, 2007; Fedoriw et al., 2012). The ability to maintain such networks also would provide flexibility in coping with environmental variation constituting a powerful selective advantage for plants as well as for animals that lack intrinsic homeostatic control (e.g., see Feil and Berger, 2007). These, as well as other consequences, promise an exciting future for the investigation of phenotypes benefiting from non-random chromosome segregation during mitosis.

## Conflict of Interest Statement

The author declares that the research was conducted in the absence of any commercial or financial relationships that could be construed as a potential conflict of interest.
